# Anti-Cancer Effect and the Underlying Mechanisms of Gypenosides on Human Colorectal Cancer SW-480 Cells

**DOI:** 10.1371/journal.pone.0095609

**Published:** 2014-04-21

**Authors:** Han Yan, Xiaobing Wang, Junfeng Niu, Yaqin Wang, Pan Wang, Quanhong Liu

**Affiliations:** Key Laboratory of Medicinal Resources and Natural Pharmaceutical Chemistry, Ministry of Education, National Engineering Laboratory for Resource Developing of Endangered Chinese Crude Drugs in Northwest of China, College of Life Sciences, Shaanxi Normal University, Xi’an, Shaanxi, China; University of Arkansas for Medical Sciences; College of Pharmacy, United States of America

## Abstract

**Background:**

Gypenosides (Gyp), the main components from *Gynostemma pentaphyllum* Makino, are widely used in traditional Chinese medicine. The present study aimed to investigate the anti-cancer effect and the underlying mechanisms of Gyp on human colorectal cancer cells SW-480.

**Materials and Methods:**

The inhibitory effect of Gyp on SW-480 cells was evaluated by MTT assay. Apoptotic cell death was detected by nuclear Hoechst 33342 staining and DNA fragmentation analysis. Apoptosis was analyzed using Annexin V-PE/7-amino-actinomycin D staining. Cell membrane integrity was evaluated with flow cytometry following PI staining. Changes of mitochondrial membrane potential (*Δψ*_m_) were detected through flow cytometry analysis of rhodamine 123 (Rh123). The role of reactive oxygen species (ROS) in Gyp induced cell death was investigated by intracellular ROS generation and general ROS scavenger. Wound-healing assay was carried out to investigate Gyp-inhibited migration of SW-480 cells *in vitro*. Additionally, the alterations in F-actin microfilaments were analyzed by FITC-labeled phalloidin toxin staining and the morphological changes were evaluated under scanning electron microscope (SEM).

**Results:**

After the Gyp treatment, the plasma membrane permeability of SW-480 cell was increased, *Δψ*_m_ was decreased significantly, the level of intracellular ROS level was increased, DNA fragmentation and apoptotic morphology were observed. Cells treated with Gyp exert serious microfilament network collapse as well as the significant decrease in the number of microvilli. Gyp induced the changes of cell viability, cell migration, intracellular ROS generation and nuclear morphology were alleviated obviously by NAC.

**Conclusion:**

The results in this study implied that ROS play an important role in Gyp induced cell toxicity and apoptosis, and the mitochondria damage may be upstream of ROS generation post Gyp treatment. The findings of the present study provide new evidences for anti-tumor mechanisms by which Gyp induces apoptosis *in vitro*.

## Introduction

Colorectal cancer (CRC) is a leading cause of death worldwide, with nearly 1,000,000 new cases and 500,000 deaths from CRC around the world every year [1], [2]. There are many risk factors for CRC, including advanced age, inflammatory bowel diseases, medical history of benign adenomatous polyps, family history of CRC, low intake of vegetables and fruits, high intake of animal fat and processed meat [3], [4].

In clinical CRC treatment, traditional therapies such as radiotherapy, chemotherapy and surgery are not the best cure method for it because of poor prognosis and serious side effects. Therefore, searching for novel anti-tumor therapeutics is extremely urgent. Now, natural medicine in cancer therapy has aroused wide concern at home and abroad, because of its safety, effiiciency and minimal side effects [5].

**Figure 1:**
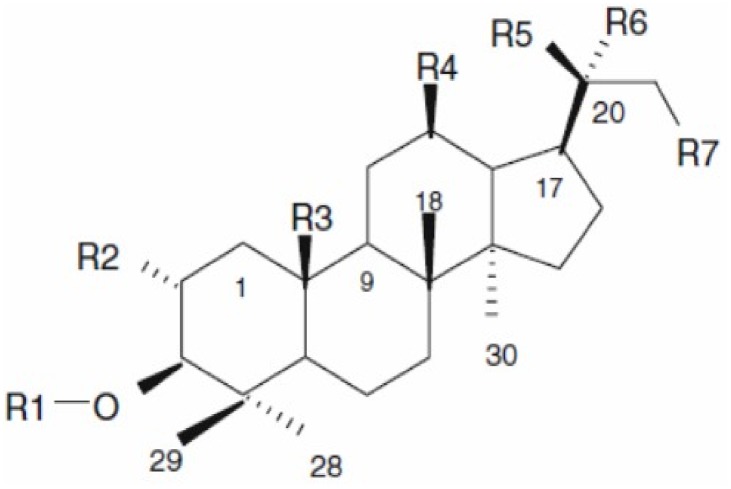
Chemical structure of dammarane skeleton of *G. pentaphyllum*.

Gypenosides (Gyp), a popular folk medicine in the China, is the major components in extracts from *Gynostemma pentaphyllum* Makino. It exist mainly as dammarane type- triterpene glycosides (Figure 1). Gyp had been known for its wide beneficial effects for treating hepatitis, hyperlipoproteinemia and cardiovascular disease[6]–[8]. Studies have shown that Gyp has an activity of anti-inflammatory, anti-thrombotic, antioxidative and anti-cancer actions [9]–[12]. But, until now, there is no report about Gyp-induced anti-tumor effect on human colorectal cancers. So, in the present study, the cytotoxicity and apoptosis of SW-480 cell induced by Gyp were investigated. Role of reactive oxygen species (ROS) in Gyp induced cell death was analyzed by intracellular ROS generation and ROS scavenger. These results may provide evidences for the role of Gyp as a potent anti-colorectal cancer agent in clinical application.

## Materials and Methods

### Chemicals and Reagents

Gyp was kindly provided by Ankang Pharmaceutical Institute of the Beijing University. The powder was dissolved in 80% ethanol (EtOH) to get a stock solution of 100 mg/ml, which was sterilized by a 0.22 µm membrane filtration. 3-(4, 5-dimethylthiazol-2-yl)-2, 5-diphenyltertrazolium bromide tetrazolium (MTT), rhodamine-123 (Rh123), Hoechst 33342, propidium iodide (PI) and N-acetylcysteine (NAC) were purchased from the Sigma Chemical Company (St. Louis, MO, USA). 2′, 7′- dichlorodihydrofluorescein-diacetate (DCFH-DA) and FITC-phalloidine were supplied by Molecular Probes Inc. (Carlsbad, CA, USA). Guava Nexin Reagent was obtained from Millipore Corporation (Billerica, MA, USA). All the other reagents were commercial products of analytical grade.

### Cell Culture

The human colon cancer SW-480 cells were obtained from the cell bank of the Chinese Academy of Science, Shanghai, China. The cell line was cultured in RPMI-1640 medium containing 10% FBS, 1% penicillin-streptomycin (100 U/ml penicillin and 100 µg/ml streptomycin) and 1% glutamine in cell culture flask under a humidified 5% CO_2_ and 95% air atmosphere at 37°C.

### Assessment of Cell Viability after Gyp Treatment

To investigate the effect of Gyp on SW-480 cell proliferation, cells were seeded in 96-well plates. Various concentrations (0, 70, 100 and 130 µg/ml; 80% ethanol was used as the solvent control) of Gyp were added and the cells were incubated for various periods of time, at a density of 1×10^5^ cells/ml, respectively. The cell viability was determined by using MTT assay [13]. The absorbance at 570 nm was recorded using a microplate reader (Bio-Tek ELX800, USA). The cell viability of Gyp treated samples was then obtained by comparing to the control.

### Flow Cytometry Analysis of Cell Membrane Integrity

PI is a fluorescent membrane-impermeant dye that stains the nuclei by intercalating between the stacked bases of nucleic acid. Since PI enters the cell only if the cell membrane becomes permeable, it is widely used in cell death research to measure the integrity of the plasma membrane. When bound to nucleic acids, the absorption maximum for PI is 535 nm and the fluorescence emission maximum is 617 nm [14]. Cells in 24-well plates were treated with the indicated concentration of Gyp for 6, 12, 24 and 48 h, respectively. Then cells were harvested and rinsed twice with PBS, stained with 5 µg/ml PI for 5 min in the dark and analyzed by flow cytometry. Data were analyzed using FCS Express V3 (De Novo Software).

### Examination of Mitochondrial Membrane Potential (Δψ_m_)

To study the *Δψ*_m_ changes, cells were stained with Rh123, which selectively enters mitochondria with an intact membrane potential and is retained in the mitochondrial [15]. Once the mitochondria membrane potential is lost, Rh123 is subsequently washed out of the cells. Cells in 24-well plates were treated with the indicated concentration of Gyp for 4 and 8 h. The cells were harvested and rinsed twice with PBS, resuspended in 500 µl of 1 µg/ml Rh123 and incubated at 37°C for 30 min in the dark. The samples were then immediately detected by flow cytometry. Data were analyzed using FCS Express V3 (De Novo Software).

### Flow Cytometry Analysis of DNA Fragmentation

To analyze DNA fragmentation, flow cytometric detection of DNA hypoploidy after adding PI to the dying cells and permeabilizing them by freeze-thawing was performed [14]. The size of DNA fragments appears as a hypoploid DNA histogram. To investigate the effect of Gyp on DNA damage of SW-480 cells, we performed oligonucleosomal DNA fragmentation by flow cytometry. Cells in 24-well plates were treated with various concentrations of Gyp for 6, 12, 24 and 48 h, respectively. Cells were then stained with 5 µg/ml PI and analyzed for DNA content by using flow cytometry.

### Hoechst 33342 Staining

In order to observe changes of nuclei morphology of tumor cells after Gyp treatment, Hoechst 33342 staining was used. After treatment with the indicated concentration of Gyp for 6, 24 and 48 h, cells were stained by 10 µM Hoechst 33342 for 15 min at room temperature. Then, the stained cells were rinsed three times with PBS and observed using a fluorescence microscope with standard excitation filters. The excitation wavelength and emission wavelength were 346 nm and 460 nm, respectively.

### Analysis of Cell Apoptosis

Cell apoptosis was detected after treatment with the indicated concentration of Gyp for 12 and 24 h. Quantification of cell apoptosis was measured by Guava Nexin assay, which utilizes Annexin V-PE to detect the phosphatidylserine on the external membrane of apoptotic cells. The membrane-impermeant dye, 7-amino-actinomycin D, is also used as an indicator of cell membrane integrity. Briefly, 100 µl cells of each sample was suspended in a mixture of 100 µl Annexin V-PE and 7-ADD binding buffer. After incubation at room temperature for 20 min, samples were analyzed by flow cytometry. The population was separated into three groups: living cells with low-level fluorescence, the apoptotic cells in earlier stages with green fluorescence, and the late apoptotic cells with both red and green fluorescence.

### Wound Healing Assay

Cells were seeded in 24-well plates and incubated 24 h. Then cells in individual wells were wounded by scratching with a pipette tip and treated with the indicated concentration of Gyp. After 24 hours incubation, the cells were photographed under phase-contrast microscopy.

### Microfilament Analysis

After different treatment, cells were fixed with 4% paraformaldehyde in PBS for 10 minutes at 37°C. Cells were permeabilized with 0.1% Triton X-100 in PBS for 7 min and blocked with 1% BSA in PBS for 1 h at 37°C. Between each step described above, cells were washed three times with PBS for 5 minutes at 37°C. Followed blocking cells were stained with 5 µg/ml FITC-phalloidine for 1 h at 37°C in the dark. Images were obtained by a laser scanning confocal microscope (TCS SP5, Leica, Germany).

### Scanning Electron Microscope (SEM) Observation

After 24 h following various treatments, cells in each group were fixed in phosphate-buffered 2.5% glutaraldehyde solution, rinsed with PBS and dehydrated by graded alcohol, critical point-dried from liquid CO_2_ and gold sputtered. The surfaces of the cells were observed by scanning electron microscope (S-3400N, Hitachi, Japan).

### Detection of Intracellular Reactive Oxygen Species (ROS) Generation and its Role in Gyp Caused Cytotoxicity

In order to determine changes in the ROS level, we measure the oxidative conversion of the sensitive fluorescent probe 2′, 7′-dichlorofluorescein-diacetate (DCFH-DA) to fluorescent 2′, 7′-dichlorofluorescein (DCF). DCFH-DA readily diffuses through the cell membrane and is enzymatically hydrolyzed by intracellular esterases to form nonfluorescent DCFH, which is then rapidly oxidized to form highly fluorescent DCF in the presence of ROS, and the fluorescence intensity is proportional to ROS production. Cells in 24-well plates were treated with the indicated concentration of Gyp for 4 and 8 h. The cells were harvested and rinsed twice with PBS, resuspended in 500 µl of 10 µM DCFH-DA and incubated at 37°C for 30 min in the dark. The samples were then immediately detected by flow cytometry. Data were analyzed using FCS Express V3 (De Novo Software).

To investigated the role of ROS in Gyp induced cell death and apoptosis, NAC (5 mM) was used as a ROS inhibitor added to culture medium prior to loading Gyp by 1 h. The cell viability decrease, intracellular ROS generation, the *Δψ*_m_ loss, the nuclear morphological changes and cell migration inhibition were specially analyzed as described above.

### Statistical Analysis

Data are expressed as mean ± standard deviation of at least three independent experiments. Statistical analysis was performed by one-way analysis of variance. Statistical significance was established at *p*<0.05.

## Results

### Effects of Gyp on Cell Viability

**Figure 2:**
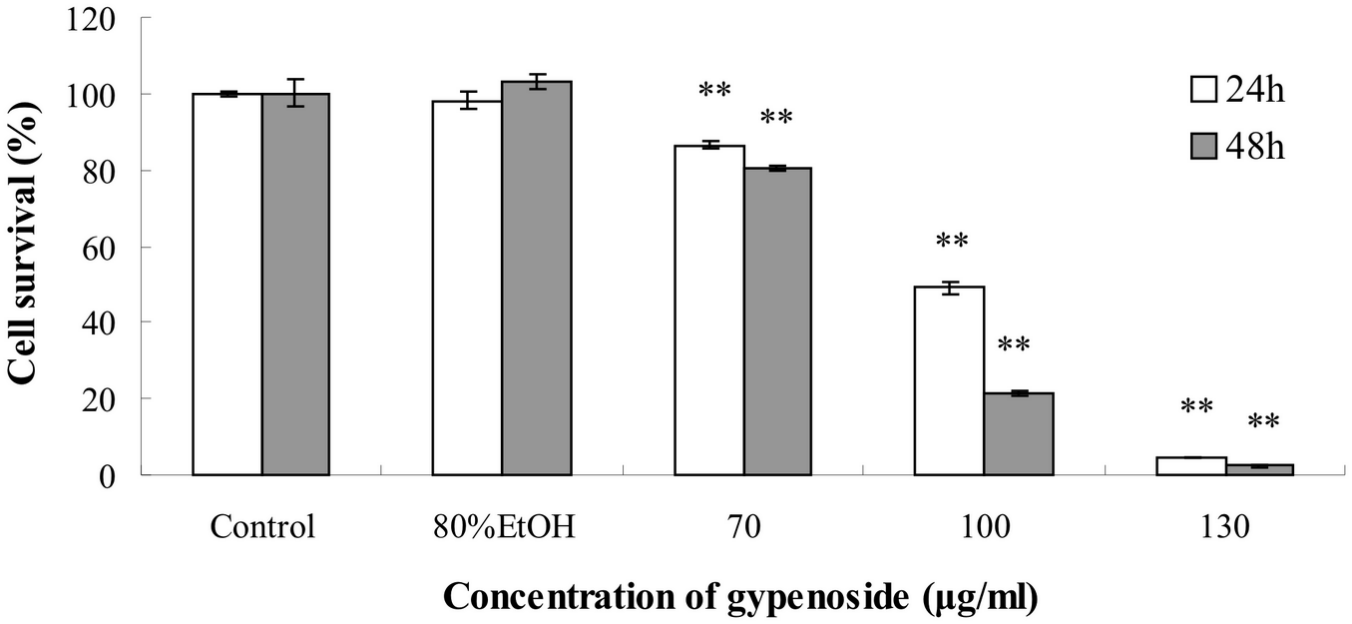
Effects of Gyp on cell viability. SW-480 were seeded in 96-well plates and treated with 0, 70, 100 and 130 µg/ml of Gyp for 24 and 48 h. Cell viability was measured by MTT assay. Each value is expressed as a mean ± S.D. of at least three independent determinations. One-way ANOVA was used for comparisons of multiple group means followed by Dunnett’s t-test. **P<0.01 versus the control. (error bars = S.D., n = 3).

Figure 2 showed that Gyp inhibited SW-480 cell proliferation in a dose- and time-dependent manner. The viability was significantly decreased when Gyp dose was 70 µg/ml or above (*p*<0.01) and the prolonged incubation enhanced the viability loss. The IC50 values were about 91.77 and 83.8 µg/ml when cells were incubated with Gyp for 24 and 48 h, respectively.

### Gyp-induced Plasma Membrane Damage of SW-480 Cells

**Figure 3:**
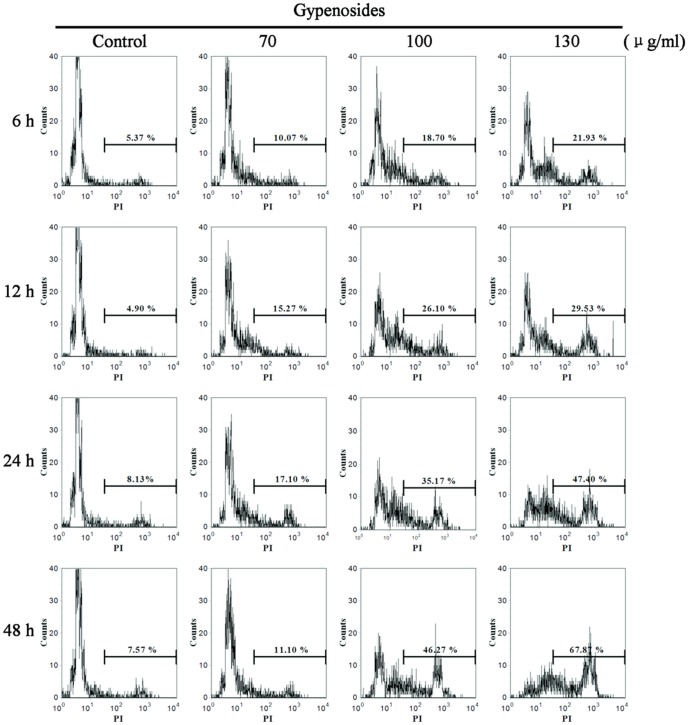
Effects of Gyp on the cell membrane integrity of SW-480 cells. Cells were treated with Gyp for 6, 12, 24 and 48(vertical axis) vs. PI fluorescence (horizontal axis).

PI staining combined with flow cytometry was used to evaluate Gyp-induced cell membrane damage. In SW-480 cells, membrane damage as an early event of Gyp induced cell injury (Figure 3). After incubation for 6 h, the percentage of cells with higher PI fluorescence gradually increased from 10.07% to 21.93% when cells were exposed to Gyp dose range from 70 to 130 µg/ml. Cells exposed to 70 µg/ml Gyp did not show much increased cell membrane damage with the prolonged incubation time. While cells after 100 µg/ml and 130 µg/ml Gyp treatment, the cell membrane damage was greatly increased by the prolonged incubation time, about 46.27% and 67.87% of cells displayed high PI fluorescence at 48 h after treatment, respectively.

### Gyp-induced the Δψ_m_ Loss in SW-480 Cells

**Figure 4:**
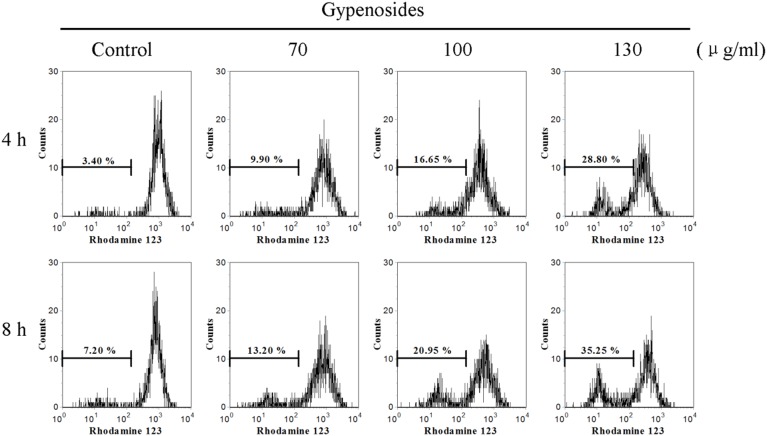
Effects of Gyp on mitochondrial membrane potential (*Δ* *ψ*_m_) of SW-480 cells. Cells were treated with Gyp for 4, 8 h and labeled with rhodamine 123 and analyzed by flow cytometry. Histograms show number of cell channel (vertical axis) vs. rhodamine 123 fluorescence (horizontal axis).

Rh123 staining combined with flow cytometry was used to evaluate Gyp-induced *Δψ*_m_ changes. As shown in Figure 4, it shows that Gyp induced the loss of *Δψ*_m_ quite early and caused the decrease of *Δψ*_m_ in a dose- and time-dependent manner. After 4 h of incubation, the percentage of cells with *Δψ*_m_ loss increased from 9.9% to 28.8% when Gyp increased from 70 µg/ml to 130 µg/ml. When the incubation time increased from 4 to 8 h, the percentage of cells with *Δψ*_m_ loss increased from 16.65% to 20.95% when Gyp was 100 µg/ml.

### Gyp-induced DNA Fragmentation of SW-480 Cells

**Figure 5:**
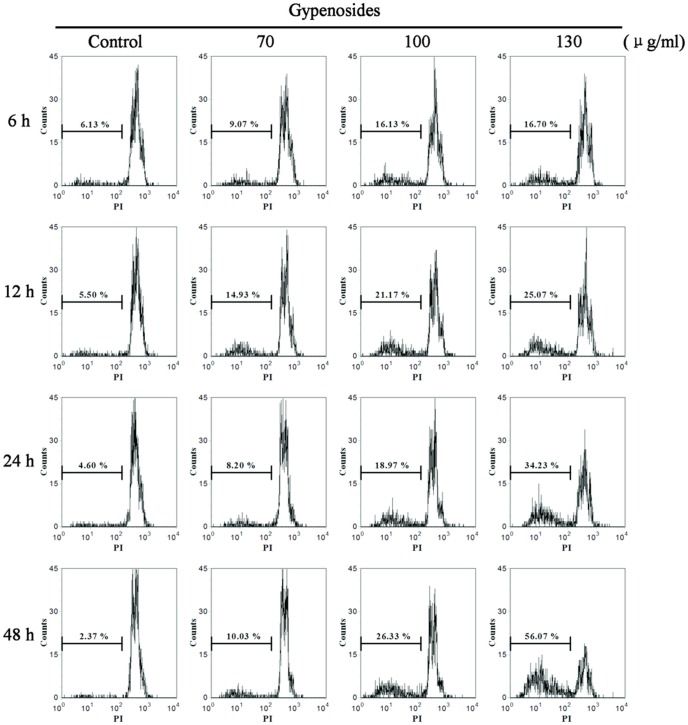
Effects of Gyp on DNA fragmentation of SW-480 cells. Cells were treated with Gyp for 6, 12, 24 and 48(vertical axis) vs. PI fluorescence (horizontal axis).

To determine the DNA damaging activity of Gyp, the percentage of damaged cells was analyzed using PI staining by flow cytometry as described in the methods. As shown in Figure 5, Gyp induced DNA fragmentation was in a dose- and time-dependent manner. No significant DNA fragmentation was detected by different dose of Gyp after treatment 6 h, while a significant amount of large-scale DNA fragmentation was observed by the higher dose Gyp (100, 130 µg/ml) after 12 h of incubation, and the DNA damage in lower Gyp dose (70 µg/ml) treated cells didn’t enhanced with the prolonged incubation time. After 48 h of incubation, the DNA fragment of control group is 2.37%, it increased to 10.03%, 26.33% and 56.07% when Gyp concertration was 70, 100 and 130 µg/ml, respectively.

### Gyp-induced Morphological Changes of SW-480 Cells

**Figure 6:**
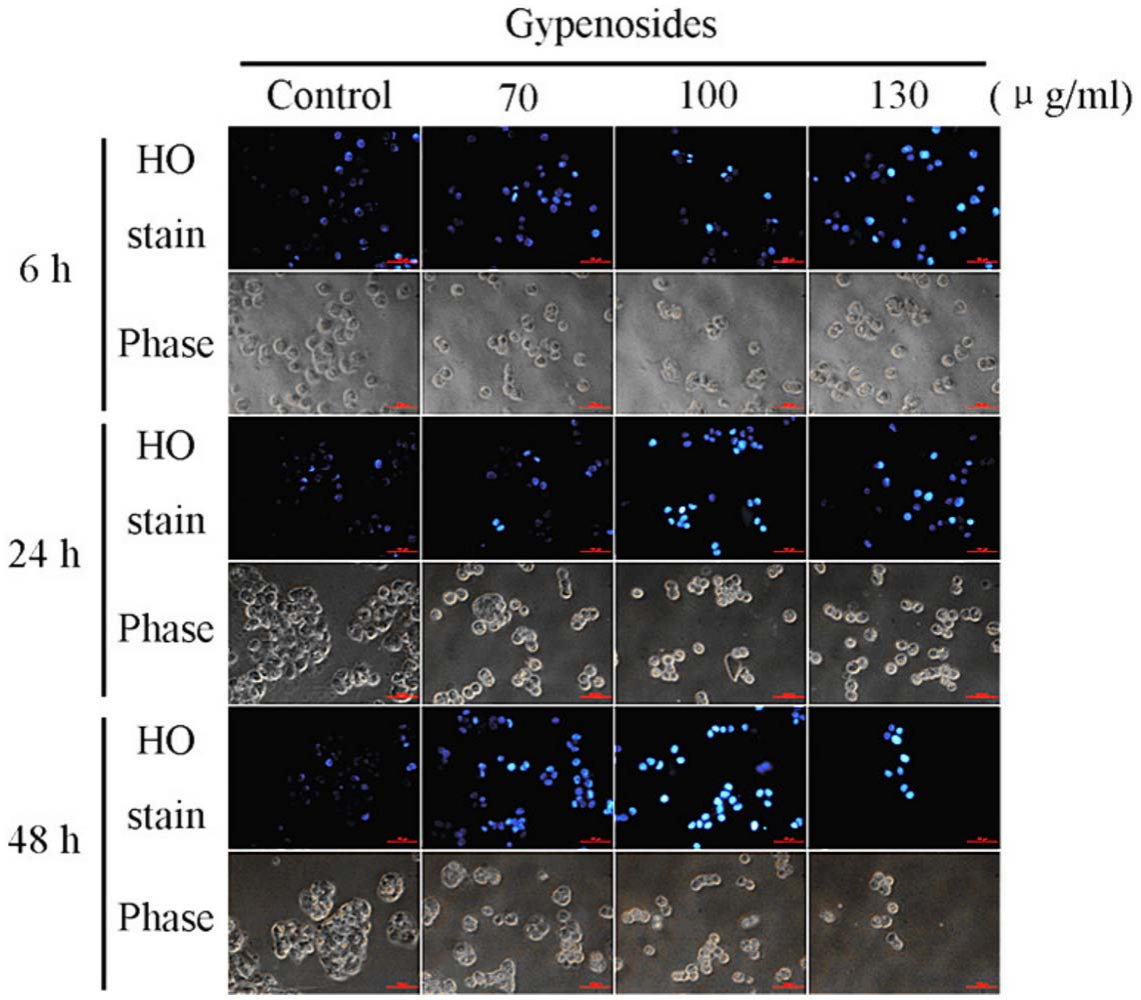
Gyp effects on the cell morphology and nucleus in SW-480 cells. Cells were treated with Gyp at the indicated concentrations for 6, 24, 48“Materials and methods”.

Figure 6 showed the results of Hoechst 33342 staining in SW-480 cells treated by Gyp. Slightly blue and homogeneous cell was observed in control group. Cells in Gyp treated groups showed enhancement of Hoechst 33342 staining and change of cell morphology in a Gyp-dose and incubation-time dependent manner. When Gyp concentration was above 100 µg/ml, SW-480 cells were seriously damaged with bright blue nuclear staining and the phase images indicated cells were shrunken to abnormal round type, and the cell number was significantly decreased.

### Detection of Cell Apoptosis using Flow Cytometry

**Figure 7:**
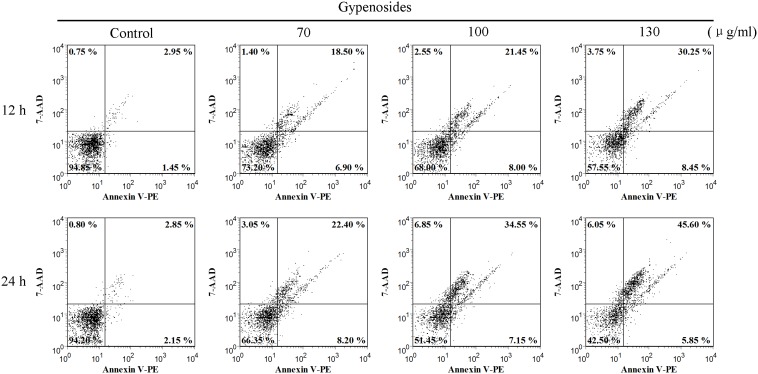
Apoptosis-inducing effect of Gyp on SW-480 cells. Dot plots of Annexin V and 7-AAD uptake after indicated concentrations in SW-480 cells. Cells were analyzed at 12, 24 h post treatment.

The apoptotic rates were measured by Annexin V-PE and 7-ADD staining after 12,24 h following various treatments. The fractions of cells in each quadrant were analyzed by quadrant statistics [16]. As shown in Figure 7, the percentages of cells with Annexin V-positive staining increased gradually in a concentration-dependent manner after Gyp treatment, suggesting that Gyp could induce apoptotic response in SW-480 cells. In untreated control group, 94.85% cells were viable, 1.45% cell population was in the early stage of apoptosis (lower right) and 2.95% cell population was in the late stage of apoptosis (upper right). While, after 12 h incubation with Gyp, the apoptotic cell population (lower right + upper right) increased from 25.40% to 38.70% when the drug concentration increased from 70 µg/ml to 130 µg/ml. When the incubation time increased from 12 to 24 h, the apoptotic cell population increased from 29.45% to 41.70% when Gyp was 100 µg/ml.

### Gyp Inhibited Migration of SW-480 In vitro

**Figure 8:**
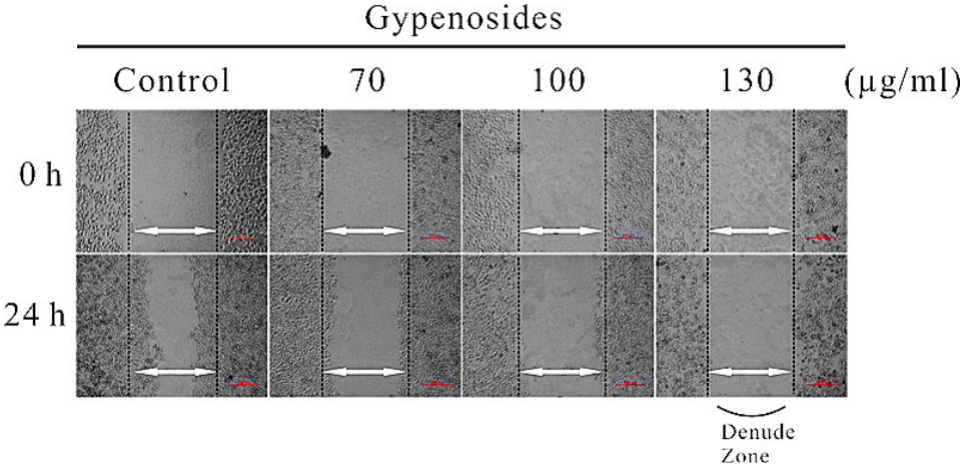
Effects of Gyp on the migration of SW-480 cells *in vitro*. Cells in 24-well plates were wounded by scratching with a pipette tip and the cells were incubated with Gyp for 24 hours. The cells were photographed under phase-contrast microscopy (×200 magnification).

To further verify the inhibitory effect of Gyp on SW-480 cell migration, a wound healing assay was conducted (Figure 8). The wound healing ability of cells reflects their movement and migration on the surface on which they are anchored to for growth. Compared with 0 h after wounding, after 24 h of incubation, very dense cells in control gradually grew to the interspace of wound, cells in 70 µg/ml Gyp treated group showed slight difference with control difference with control, while cells in 100 µg/ml and 130 µg/ml Gyp treated groups rarely grew to the interspace of wound, and the cell density was seriously decreased.

### Microfilament Analysis

**Figure 9:**
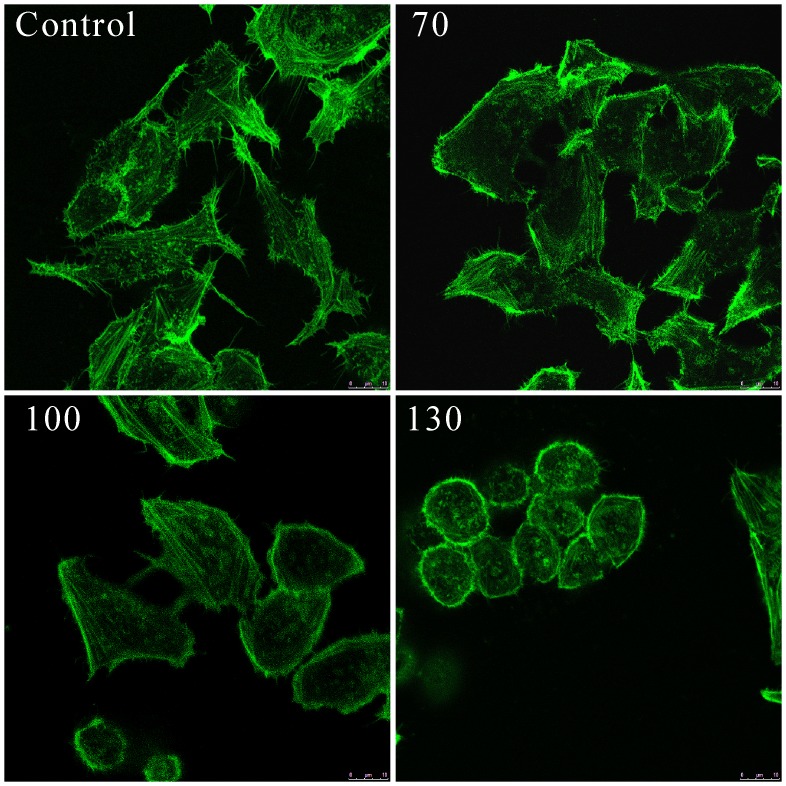
Changes in the actin cytoskeleton of SW-480 cells after Gyp. Cells were stained with phalloidin (green) for F-actin. Scale bars, 10 µm.

It is well established that cytoskeleton elements were closely related to cell movement [17], [18]. The changes of F-actin microfilaments organization in SW-480 cells after Gyp treated was investigated by fluorescence microscopy using FITC-labeled phalloidin toxin. As shown in Figure 9, control cells showed a regular array of defined actin filaments present along the cells, evenly distributed in the cytoplasm, while cells in 100 µg/ml Gyp showed a disorganization of actin filaments, a increase of actin stree fibers and green fluorescence spots were observed. Cells treated with 130 µg/ml Gyp demonstrated an absolute damage of actin network and complete disappearance of actin filaments.

### SEM Observation

**Figure 10:**
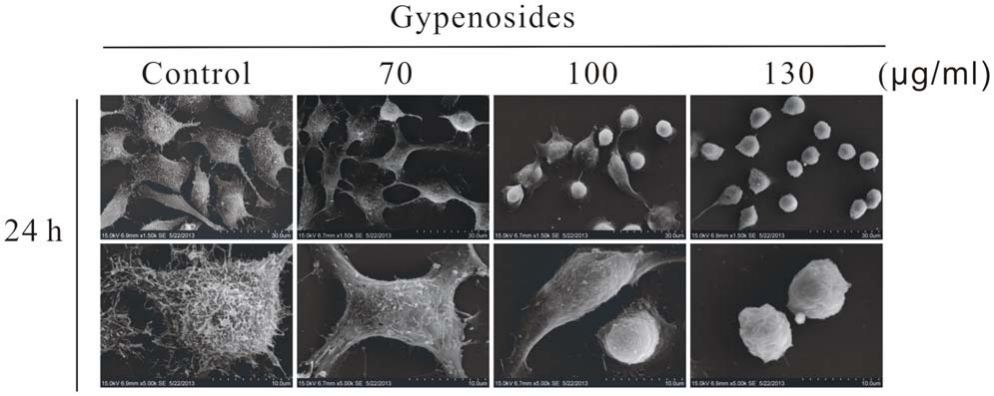
Scanning electron microscopic images of SW-480 cells at 24 h after Gyp. Magnification of the images was 1500 and 5000×, respectively. Scale bars: 30, 10 µm.

The morphological changes were observed under SEM (Figure 10). In control group, cells appeared epithelial in shape with numerous microvilli over the surface of the cell. While in 70 µg/ml, cells showed a significant decrease in the number of microvilli, the surface of many cells becoming relatively smooth with no obvious microvilli. When Gyp dose was above 100 µg/ml, SW-480 cells were seriously damaged with apparent deformation, shrunken to abnormal round type, and the cell number was significantly decreased. While in 130 µg/ml, some papillous protuberances were observed on the surface of cells where the cytoplasm seemed to have extruded through the membrane boundary.

### ROS Involved in Gyp-induced SW-480 Cytotoxicity

**Figure 11:**
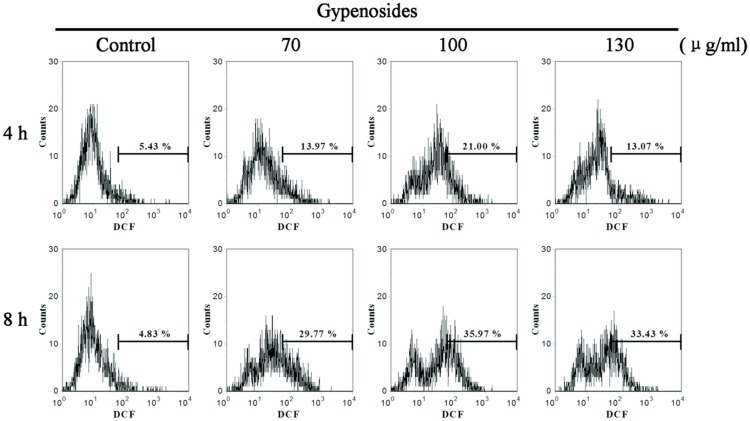
Gyp-induced production of reactive oxygen species (ROS) in SW-480 cells. Cells were treated with Gyp for 4, 8–DA and the fluorescence intensity of the oxidized product DCF in individual cells was detected by flow cytometry. The percentage of fluorescent cells in each group was shown.

The intracellular ROS production was analyzed by flow cytometry with DCFH-DA staining. The data shown in Figure 11 suggest the intracellular ROS levels was increased after Gyp treatment, at 4 h after treatment, there were about 13.97%, 21% and 13.07% of cells in 70, 100 and 130 µg/ml Gyp treated groups showed bright DCF fluorescence, while only 5.43% of cells in control group showed bright DCF fluorescence. When the incubation time increased from 4 to 8 h, the percentage of cells with bright DCF fluorescence increased to 29.77%, 35.97% and 33.43% when Gyp was 70, 100 and 130 µg/ml, respectively. We found the ROS generation was first increased with increasing Gyp concentration and then slightly decreased at much higher Gyp dose, which may due to more cell lysis caused by Gyp treatment at higher drug concentration.

**Figure 12:**
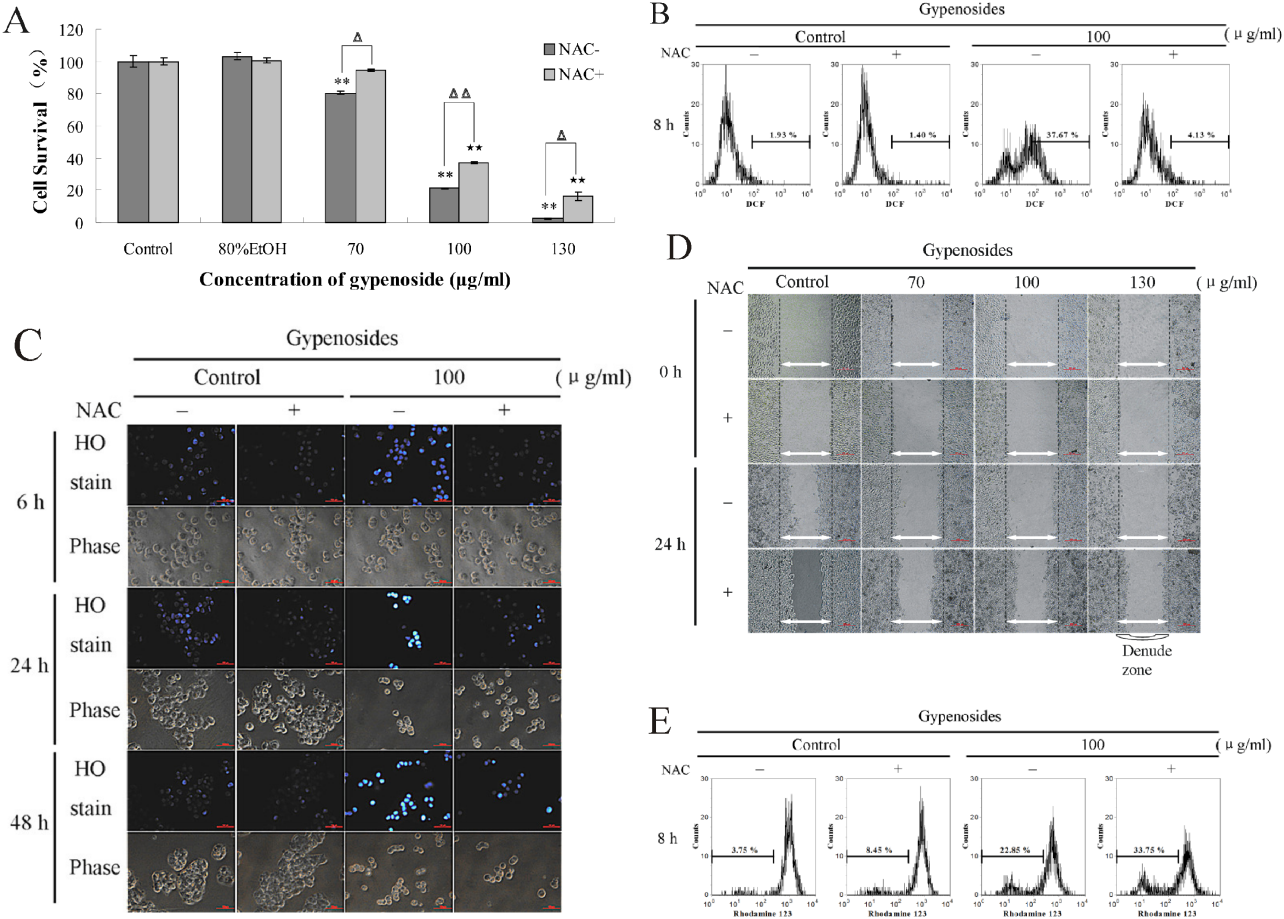
Effect of ROS scavenger NAC (N-acetylcysteine) on the effect of Gyp on SW-480 cells. (A) Cell viability, (B) intracellular ROS generation, (C) nuclear morphological changes, (D) cell migration, (E) *Δψ*_m_ loss were specially analyzed as described in “Materials and methods”.

To further examine if intracellular ROS elevation was involved in Gyp-induced cell death, we performed subsequent assays. Result in Figure 12A shows that the decreased cell viability caused by Gyp was greatly rescued by NAC (*p*<0.05). In addition, the Gyp caused intracellular ROS generation, chromatin condensation and cell migration inhibition were all visibly prevented by NAC (Figure 12B–D). However, the decreased *Δψ*_m_ caused by Gyp was not prevented by NAC (Figure 12E). These results indicate that ROS was involved in Gyp-induced damage in SW-480 cells and the *Δψ*_m_ loss may be an important upstream activator of ROS generation, and the increased ROS might be stimulated by the damaged mitochondria.

## Discussion

Cancer is a complex disease and the traditional cancer therapies have not progressed significantly in the last few years. The major limitations of surgery, chemotherapy and radiotherapy are that they cause serious side effects and poor prognosis due to toxicity of non-cancerous tissue and chemoresistance [19]. However, natural medicine has aroused wide concern in the area of cancer chemotherapy because of safety, efficacy and anti-multidrug resistance.

Gyp has been used as traditional Chinese herbal medicine for hundreds of years because of its variety of health benefits [20]–[22]. In the present study, Gyp induced apoptosis in human colorectal cancer SW-480 cells was investigated.

The results showed that Gyp decreased the percentage of viable cells in SW-480 cells. Meanwhile, previous studies have demonstrated that the cytotoxic effect of Gyp on normal PBMC cells was much less [23]. The results suggest that Gyp is capable of exerting different alternative cytotoxicity in cancer cells and normal cells, which might be potentially useful as a cancer preventive or treatment agent.

The cell line SW-480, established from the primary adenocarcinoma arising in the colon, was Duck’s stage B (Dukes’ B means the cancer has grown through the muscle layer of the colon or rectum, invaded through bowl wall but lymph nodes clear) [24]. Spread is through local invasion through the bowl wall and via local lymphatics, blood (portal vein into liver) and transcoelomic. Therefore, metastasis is one of the major challenges for a successful cancer treatment [25] and the prevention of cancer metastasis is as important target for improving a patient’s prognosis. In this study, we investigated whether Gyp could inhibit the migration of SW-480 cells. Results in Figure 8 suggested Gyp inhibited the migration of the cell type in a Gyp dose dependent manner. The actin cytoskeleton is a structural network of proteins that are essential for multiple biological functions including cell contraction, cell motility and vesicle trafficking et al [26], [27]. The changes in cellular compartments may affect the dynamics of the cell adhesion [18]. Figure 9 exposed the changes of microfilament, disordered arrangement of F-actin and complete disappearance of microfilament network was observed in 100, 130 µg/ml Gyp. Besides, as shown in figure 10, the number of microvilli was significantly decreased when Gyp dose was 70 µg/ml or above. The results suggest Gyp induce microfilament network collapse, and eventually, injure the cell shape and migration ability.

Apoptosis plays an important role in homeostasis and can be induced and regulated by many signal stimulus pathways [28], [29]. Dysregulation of apoptosis is considered as a major cancer hallmark, so that apoptosis induction is arguably the most potent defence against cancer [30], [31]. Many studies have revealed that various compounds extracted from plants could induce apoptosis in many cancer cells [29], [32], [33]. DNA fragmentation and some morphological characteristics including membrane blebbing, cellular shrinkage, chromatin condensation and formation of apoptotic bodies are typical biochemical feature of apoptosis [34], [35]. In present study, Gyp was able to cause obvious DNA fragmentation in SW-480 cells in a dose- and time-dependent manner. Besides, Hoechst 33342 staining assay displayed some morphological characteristics after Gyp treatment such as cell shrinkage, chromatin condensation with margination of chromatin to the nuclear membrane and fragmented punctate blue nuclear fluorescence in SW-480 cells. Finally, figure 7 suggest the Gyp could significantly increase the apoptotic cells and decrease the viable cells, indicating the apoptotic response was markedly potentiated after Gyp in SW-480 cells. Therefore, the results indicate Gyp could induce apoptosis in SW480 cells.

The damage of cell membrane system will cause membrane depolarization, disturbe asymmetry of membrane’s lipids, induce inhibition of membrane enzymes, cause lost of plasmatic membrane integrity [36], and these cellular functions may eventually induce cell apoptosis by the engagement of death receptors [37]. Staining with PI and analyzed by flow cytometry demonstrated that Gyp caused cell membrane damage was an early event. We speculate that the mechanism of gypenosides in plasma membrane damage perhaps due to the aglycones of the gypenosides [38] have its site of action on cell membrane, thereby damaging cell membrane or entering cells to prevent tumor growth.

The mitochondria play an important role in the progression of apoptosis [39]. Therefore, mitochondria membrane potential changes after different concentration of Gyp treatment in SW-480 cells was measured. In the study, the decrease of *Δψ*_m_ was at early 4 h after Gyp treatment and enhanced by increasing concentration of Gyp, suggesting Gyp induced cell apoptosis in SW-480 cells might be mitochondria dependent.

Besides, Enhancement of ROS production has been associated with the apoptotic response induced by several pro-apoptotic compounds [40]. Our results showed Gyp treatment significantly stimulated ROS generation in SW-480 cells. NAC, a scavenger of ROS, was used to confirm the effect of ROS after Gyp treatment. NAC significantly rescued Gyp induced SW-480 cytotoxicity, intracellular ROS generation, nuclear morphological changes and cell migration inhibition, but not the *Δψ*_m_ decrease, implying ROS played an important role in Gyp induced SW-480 cell toxicity and cell apoptosis, and the ROS generation was downstream of mitochondria damage post Gyp treatment, and the increased ROS production might be stimulate by the damaged mitochondria in our experimental system.

In conclusion, the present study evaluated the cytotoxicity of Gyp in SW-480 cells, demonstrated that Gyp could cause cell membrane integrity damage, decrease the *Δψ*_m_ level, induce DNA fragmentation and initiate apoptotic response in SW-480 cells. ROS generated in SW-480 cells play an important role in Gyp induced cell death. And, it is speculated that Gyp induced cell apoptosis in SW-480 cells might be death receptors pathway and mitochondria dependent. In addition, our study also showed that Gyp could exert an inhibitory effect on cell migration *in vitro* and serious microfilament network collapse as well as the significant decrease in the number of microvilli. These results suggest Gyp induce microfilament network collapse and injure the cell shape and migration ability. These studies suggest Gyp may have great value in human colorectal cancer treatments. Further investigations are needed to explain the molecular mechanisms of Gyp in cancer therapy.
